# A G-protein-biased S1P_1_ agonist, SAR247799, improved LVH and diastolic function in a rat model of metabolic syndrome

**DOI:** 10.1371/journal.pone.0257929

**Published:** 2022-01-14

**Authors:** Maria Francesca Evaristi, Bruno Poirier, Xavier Chénedé, Anne-Marie Lefebvre, Alain Roccon, Florence Gillot, Sandra Beeské, Alain Corbier, Marie-Pierre Pruniaux-Harnist, Philip Janiak, Ashfaq A. Parkar

**Affiliations:** 1 Diabetes and Cardiovascular Research, Sanofi R&D, Chilly-Mazarin, France; 2 Molecular Histology and Bioimaging Translational Sciences, Sanofi R&D, Chilly-Mazarin, France; 3 Biomarkers and Clinical Bioanalyses, Translational Medicine and Early Development, Sanofi R&D, Montpellier, France; 4 Diabetes and Cardiovascular Research, Sanofi US Services, Bridgewater, NJ, United States of America; University Medical Center Utrecht, NETHERLANDS

## Abstract

**Aim:**

Heart failure with preserved ejection fraction (HFpEF) is a major cause of death worldwide with no approved treatment. Left ventricular hypertrophy (LVH) and diastolic dysfunction represent the structural and functional components of HFpEF, respectively. Endothelial dysfunction is prevalent in HFpEF and predicts cardiovascular events. We investigated if SAR247799, a G-protein-biased sphingosine-1-phosphate receptor 1 (S1P_1_) agonist with endothelial-protective properties, could improve cardiac and renal functions in a rat model of metabolic syndrome LVH and diastolic function.

**Methods:**

31- and 65-week-old obese ZSF1 (Ob-ZSF1) rats, representing adult and aged animals with LVH and diastolic dysfunction, were randomized to a chow diet containing 0.025% (w/w) of SAR247799, or control (CTRL) chow for 4 weeks. Age-matched lean ZSF1 (Le-ZSF1) rats were fed control chow. Echocardiography, telemetry, biochemical and histological analysis were performed to evaluate the effect of SAR247799.

**Results:**

Echocardiography revealed that Ob-ZSF1 rats, in contrast to Le-ZSF1 rats, developed progressive diastolic dysfunction and cardiac hypertrophy with age. SAR247799 blunted the progression of diastolic dysfunction in adult and aged animals: in adult animals E/e’ was evaluated at 21.8 ± 1.4 for Ob-ZSF1-CTRL, 19.5 ± 1.2 for Ob-ZSF1-SAR247799 p<0.01, and 19.5 ± 2.3 for Le-ZSF1-CTRL (median ± IQR). In aged animals E/e’ was evaluated at 23.15 ± 4.45 for Ob-ZSF1-CTRL, 19.5 ± 5 for Ob-ZSF1-SAR247799 p<0.01, and 16.69 ± 1.7 for Le-ZSF1-CTRL, p<0.01 (median ± IQR). In aged animals, SAR247799 reduced cardiac hypertrophy (g/mm mean ± SEM of heart weight/tibia length 0.053 ± 0.001 for Ob-ZSF1-CTRL vs 0.046 ± 0.002 for Ob-ZSF1-SAR247799 p<0.01, Le-ZSF1-CTRL 0.035 ± 0.001) and myocardial perivascular collagen content (p<0.001), independently of any changes in microvascular density. In adult animals, SAR247799 improved endothelial function as assessed by the very low frequency bands of systolic blood pressure variability (mean ± SEM 67.8 ± 3.41 for Ob-ZSF1-CTRL 55.8 ± 4.27 or Ob-ZSF1-SAR247799, p<0.05 and 57.3 ± 1.82 Le-ZSF1-CTRL), independently of any modification of arterial blood pressure. In aged animals, SAR247799 reduced urinary protein/creatinine ratio, an index of glomerular injury, (10.3 ± 0.621 vs 8.17 ± 0.231 for Ob-ZSF1-CTRL vs Ob-ZSF1-SAR247799, respectively, p<0.05 and 0.294 ± 0.029 for Le-ZSF1-CTRL, mean ± SEM) and the fractional excretion of electrolytes. Circulating lymphocytes were not decreased by SAR247799, confirming lack of S1P_1_ desensitization.

**Conclusions:**

These experimental findings suggest that S1P_1_ activation with SAR247799 may be considered as a new therapeutic approach for LVH and diastolic dysfunction, major components of HFpEF.

## 1. Introduction

Heart failure with preserved ejection (HFpEF) affects approximately 50% of heart failure patients and, unlike heart failure with reduced ejection fraction, there are no drugs approved for HFpEF. Accompanied with the rise in life-expectancy, the prevalence of HFpEF continues to increase and its prognosis is bleak with a 5 year mortality rate over 50% [1, 2]. Multiple clinical trials have investigated approved drugs in heart failure with reduced ejection fraction in the context of HFpEF with disappointing results [3, 4]. Only very recently, the EMPEROR-Preserved clinical trial has shown that empagliflozin reduced cardiovascular death or hospitalization in HFpEF patients [5]. HFpEF is a complex syndrome in which patients present with multiple comorbidities including hypertension, diabetes, obesity, chronic kidney disease and atrial fibrillation [6]. In addition to left ventricular diastolic dysfunction, these multiple risk factors result in left ventricular hypertrophy (LVH) that contributes to vascular dysfunction and increased myocardial stiffness.

In order to bring forward effective treatments for HFpEF patients, there is a need to develop new drugs tailored to the pathophysiology of HFpEF *per se*. Left ventricular diastolic dysfunction plays a fundamental role in the pathophysiology of HFpEF.

Many studies have documented that endothelial dysfunction is prevalent in HFpEF patients, particularly in the microvasculature, and it is present in multiple vascular beds including the coronary, pulmonary, renal, or skeletal muscle circulations [7]. Furthermore, established endothelial dysfunction predicts poor prognosis in HFpEF patients [7]. According to the paradigm from Paulus and Tschope [8], the high prevalence of these comorbidities are responsible for a systemic proinflammatory state associated with endothelial dysfunction that causes reduced nitric oxide bioavailability and paracrine signaling changes in the cardiomyocyte, namely decreased cyclic guanosine monophosphate with subsequent titin hyper-phosphorylation and an increase in cardiomyocyte resting tension. Other consequences of endothelial dysfunction are reduced coronary flow reserve leading to myocardial ischemia, and transmigration of monocytes into the myocardium. All these changes are believed to cause concentric hypertrophy, cardiac stiffening, interstitial fibrosis and diastolic dysfunction [6].

Sphingosine 1-phosphate (S1P) is a lipid mediator secreted by endothelial cells, red blood cells and platelets, and exerts its effects through five receptors (S1P_1-5_) [9]. Sphingosine-1-phosphate receptor 1 (S1P_1_) has the most prominent role on the endothelium, regulates vascular tone, inflammatory cell adhesion and migration, and endothelial cell barrier function [10]. High S1P concentrations are found in the plasma of healthy individuals where it is carried primarily by HDL. HDL-bound S1P, acting through S1P_1_, limits vascular inflammation and atherosclerosis [11]. Reduced plasma S1P and dysfunctional HDL are both found in a variety of patient populations with endothelial dysfunction. Plasma S1P is reduced in patients with systolic heart failure due to ischemic heart disease [12], in type 2 diabetic patients [13] as well as in rodent models of post-ischemic heart failure [14]. Although several S1P_1_ modulators are either approved in multiple sclerosis (e.g. fingolimod, siponimod and ozanimod) or are in clinical development for immuno-inflammatory disorders, they were all developed as S1P_1_ functional antagonists that cause peripheral blood lymphopenia through S1P_1_ desensitization. In contrast, SAR247799 is a G-protein-biased S1P_1_ agonist, preferentially activating G-protein over β-arrestin and internalization signaling pathways and activates S1P_1_ without causing desensitization [15]. SAR247799 thus exhibits endothelial-protective properties without reducing lymphocytes [16]. SAR247799 improves the coronary microvascular hyperemic response in pigs, improves endothelial barrier integrity in a rat model of renal ischemia/reperfusion injury [16] and restores renal function and endothelial parameters in a rat model of type-2 diabetes [17]. Moreover, SAR247799 demonstrates good safety and tolerability in healthy subjects [18] and improves endothelial function, as assessed by flow-mediated dilation in type-2 diabetic patients [17]. The processes causing endothelial dysfunction are closely intertwined with cardiovascular disease and heart failure. Despite efforts to explore the effects of some endothelial-based therapies in the components of HFpEF, there is no confirmation at present that restoring endothelial function would provide clinical improvements in the progression of the components of HFpEF. We therefore set out to test whether 4-week oral treatment with the S1P_1_ agonist, SAR247799, a molecule that we have previously shown has endothelial protective effects in animals and humans [15–18] improves cardiac structural and functional parameters in a rat model of LVH and diastolic function. The dose and the duration of treatment were chosen based on previous studies with SAR24799 in diabetic patients and diabetic rats where SAR247799 showed a significant effect on maximum flow mediated dilation change in diabetic patients and on protein/creatinine ratio and endothelial biomarkers in diabetic rats [17]. We used the diabetic Zucker fatty spontaneously hypertensive heart failure F1 hybrid (ZSF1) rat, carrying two separate leptin receptor mutations (fa and facp), which has been described as a genetically-hypertensive, obese and diabetic model of HFpEF [19, 20]. ZSF1 rats have been described as a good model of HFpEF [19]. Indeed, these rats present with the same risk factors found in HFpEF patients *i*.*e*. hypertension, obesity and type-2 diabetes and develop concentric LVH, diastolic dysfunction with preserved systolic function, pulmonary congestion and exercise intolerance, miming the features of human HFpEF [21]. Although the ZSF1 rats do not reproduce the slow onset seen in patients with hypertension that lead to HFpEF development, this rat model may be argued to mimic the HFpEF patient in many scenarios [21], including the systemic proinflammatory state associated with endothelial dysfunction and was, thus, used in this study. As HFpEF patients are heterogeneous with respect to clinical criteria, we tested the effect of SAR247799 in two cohorts of obese ZSF1 rats that differed by age (termed adult and aged animals), and consequently the degree of diastolic dysfunction and hypertrophy.

## 2. Methods

### 2.1. Animals

**Fig 1:**
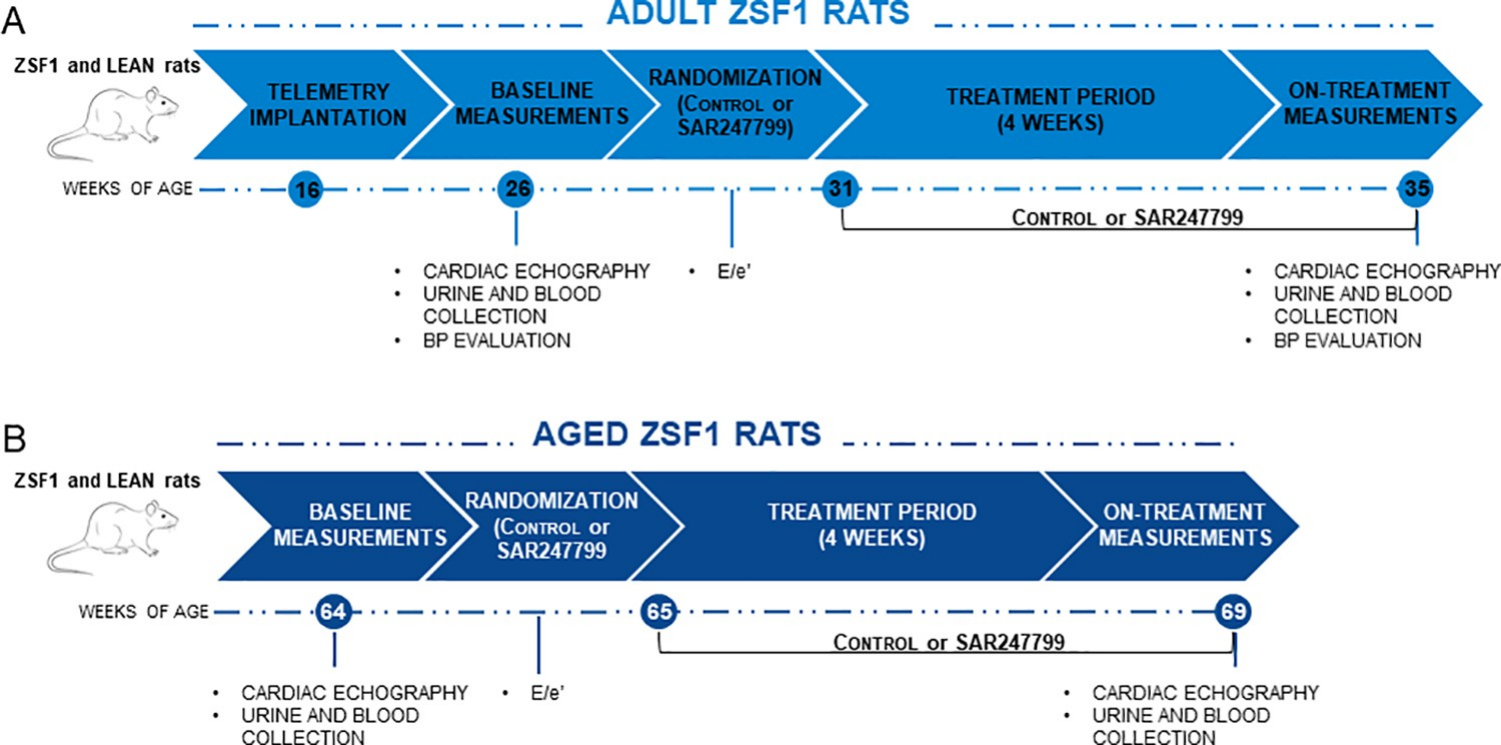
Overview of studies for adult and aged ZSF1 rats. Ob-ZSF1 rats were randomized on E/e’ parameter from doppler echocardiographic analysis. For adult animal study, Le-ZSF1-CTRL N = 9, Ob-ZSF1-CTRL N = 11 and Ob-ZSF1-SAR247799 N = 11. For aged animals study Le-ZSF1-CTRL N = 9, Ob-ZSF1-CTRL N = 7 and Ob-ZSF1-SAR247799 N = 7; N = number of rats.

Male obese ZSF1 (Ob-ZSF1) and Lean ZSF1 (Le-ZSF1) rats were purchased from Charles River (USA). Only male animals were considered in the present study, as at the time of the experiments only male ZSF1 rats were known to develop cardiac dysfunction. Two cohorts were obtained of different ages and considered for adult and aged animals protocols—as detailed below and in **Fig 1**. All animals were housed in groups of two in a climate-controlled environment (21–22°C, 55% humidity) with a 12:12 h light-dark cycle and provided with diet and water ad libitum. Control diet A04 (SAFE) was used from the arrival of the animals up to the starting of the experimental protocols. All animal studies were performed in accordance with the European Community standard on the care and use of laboratory animals and approved by the IACUC of Sanofi R&D. A lower number of lean animals was considered at the beginning of this study as lean rats were included as a comparison group to confirm the presence of LVH and diastolic dysfunction in ZSF1 rats and in order to follow the principle of reduction of 3Rs. At the end of each experiment, all animals were anesthetized under 3% isoflurane and euthanized by an intravenous injection of supersaturated KCl. All experiments were performed blind with respect to the treatment (blinded experimenters for control or treated Ob-ZSF1 rats), while the group Le-ZSF1 was not possible to be blinded due to the lower body weight of this group.^

### 2.2. In vivo experimental protocols

#### 2.2.1. Adult ZSF1 rats

10-12-week-old rats (22 Ob-ZSF1 and 9 Le-ZSF1 rats) were purchased and, 4-weeks after arrival, were implanted with a telemetry device (Data Sciences International™), as described below. At 26 weeks of age, rats underwent a baseline echocardiography, blood and urine collection. Ob-ZSF1 rats were randomized based on the diastolic function parameter E/e’ by SAS v9.4. SAR247799 was incorporated at the dose of 0.025% (w/w) in control chow (diet reference 1534–00 SSNIFF Spezialdiäten GmbH) by the manufacturer and was stored in cool and dry condition throughout the duration of the study. This control chow was composed by 58% carbohydrates, 33% protein and 9% fat. Animals were fed during the study with control chow formulated with SAR247799 (Ob-ZSF1-SAR247799 N = 11) or with control chow (Ob-ZSF1-CTRL N = 11). Le-ZSF1 rats were maintained on control chow. Post treatment assessments were made 4 weeks later by echocardiography, as well as blood and urine collection.

#### 2.2.2. Aged ZSF1 rats

5-7-week-old rats (22 Ob-ZSF1 and 9 Le-ZSF1, rats) were purchased and kept in the animal facilities up to 64 weeks of age, when baseline echocardiography was performed, and samples of blood and urine collected on the animals alive (14 Ob-ZSF1 and 9 Le-ZSF1). 8 Ob-ZSF1 rats dead starting from 60 weeks of age due to end-stage renal failure. Ob-ZSF1 rats were randomized based on the E/e’ value to a control chow diet (Ob-ZSF1-CTRL N = 7) or to a diet with chow supplemented with 0.025% (w/w) SAR247799 (Ob-ZSF1-SAR247799 N = 7), (SSNIFF Spezialdiäten GmbH). Le-ZSF1 rats were maintained on control chow. Post-treatment echocardiography was performed 4 weeks after randomization, at which point urine and blood were also collected. Histological analyses were performed on cardiac tissues as detailed below.

### 2.3. Telemetry measurements

In order to evaluate blood pressure and systolic blood pressure (SBP) variability (frequency-domain analysis), rats included in the adult animal group were equipped with a telemetry device. Rats were anesthetized with 4.5% isoflurane, which was then progressively lowered and maintained at 2% until the end of the surgery. Rats were placed on thermostatically-controlled heating pads, and body temperature was maintained at 37°C. Animals were then administered several drugs: a central analgesic (fentanyl at 10 μg/kg, SC), a mixture of local analgesics (lidocaine at 2 mg/kg and bupivacaine at 2 mg/kg, on surgery site, SC), an anti-inflammatory (carprofen at 4 mg/kg, SC) and an antibiotic (long acting terramycine at 30 mg/kg IM the day of surgery and 3 days later). Rats were implanted with HD-S11 devices, placed in the abdomen, for BP measurement, which was recorded through a catheter inserted into the abdominal aorta. At the end of the surgery, after recovery from anesthesia in a temperature-controlled ventilated room, rats received buprenorphine (10 μg/kg, SC) and were housed in separate cages for 10 days, during which they received carprofen and buprenorphine (4 mg/kg and 10 μg/kg respectively, SC) daily on the first 2 days. Rats were subsequently housed in groups of two per cage.

Telemetry device data were collected using Hem 4.3 acquisition software (Notocord Systems) connected to DSI’s telemetry device. At baseline and 4 weeks after treatment initiation, diastolic and systolic blood pressures were recorded continuously for 24 hours starting at 11 am. For the frequency domain analysis, SBP data points were re-sampled at 8 Hz. Periods of 512 points without erratic fluctuations (due to interruptions or interferences derived from animal movements) were visually identified and used for the calculation of the SBP variability.

Fast Fourier transformation was performed, and power spectra of each period were extracted using Hem 4.3. The software was set to determine very low frequencies (VLF: 0.02–0.2 Hz), low frequencies (LF 0.2–0.6 Hz) and high frequencies (HF: 0.6 to 4 Hz) spectral powers. Spectral powers are the area under the curve of the power spectra in the respective frequency bands. VLF are expressed in normalized units (n.u.) (i.e. the percentage of the VLF power with respect to the sum of the VLF, LH and HF powers) [22].

### 2.4. Echocardiography

To evaluate diastolic and systolic function and left ventricular geometry, transthoracic cardiac echography was performed. Rats were anesthetized in an induction cage with 3% isoflurane and then maintained to steady-state sedation level with 2% isoflurane by the nose connected to the anesthesia system. The desired depth of anesthesia was confirmed in animals by the absence of reflex after toe pinch. Rats were placed in a decubitus position on a warming pad, the chest was shaved, and hair removal cream was used to remove the remaining body hairs. A gel (Ocry-gel, TVM) was applied to both eyes to prevent drying of the sclera and a lubricated rectal probe was inserted to continuously monitor body temperature in order to maintain it in a range of 37°C ± 0.5. ECG was recorded from suitable electrodes connected to Affiniti 50 (Philips). A layer of preheated gel was applied over the chest. Two-dimensional, M-mode and pulse wave Doppler were obtained from a 15-MHz ultrasound probe, while tissue Doppler at mitral annulus level was recorded from a 12-MHz ultrasound probe. Video were recorded and then analyzed off-line in Q-Station (Philips). All measures were taken from five cardiac cycles and then averaged.

### 2.5. Plasma and urinary evaluations

18-hour urine samples were collected in metabolic cages (Tecniplast). Urinary protein, creatinine, chloride and sodium concentration were measured using a PENTRA 400 analyzer (Horiba). Blood samples were collected in K_2_-EDTA tubes from the caudal vein in animals under 1.5–2% isoflurane anesthesia. Blood lymphocyte count was measured by MS9-5 (Melet Schloesing) in freshly collected blood, and then blood samples were immediately centrifuged at 4°C/10 min/10,000g before storing plasma at -80°C. SAR247799 concentration was measured in 4-week post-treatment plasma samples by a qualified liquid chromatography tandem mass spectrometry method using a Shimadzu chromatographic system coupled to an API 4000 mass spectrometer (Sciex). Fructosamine, creatinine, chloride and sodium concentration were measured using a PENTRA 400 analyzer. Fractional excretion of sodium (FENa) and chloride (FECl) were then calculated by (urinary sodium / plasma sodium)*(plasma creatinine/ urinary creatinine)*100 and (urinary chloride / plasma chloride)*(plasma creatinine/ urinary creatinine)*100. Daily food intake was recorded in adult and aged animals.

### 2.6. Collagen content and microvascular quantification

Transversal 5-μm-thick sections were cut from formalin-fixed and paraffin-embedded heart slices and mounted onto microscope slides. Masson’s trichrome stain was used for collagen content quantification. Immunohistochemical staining of vessels was carried out using the Ventana Discovery XT automated system with recommended reagents (Ventana Medical Systems Inc). Tissue slides were dewaxed and pretreated with cell-conditioning buffer CC1 at 95°C for 48 minutes. An anti-CD31 antibody (ab182981, Abcam) was used at 5 μg/mL final concentration for 1 hour at 24°C following by goat anti-rabbit IgG biotin conjugate used as secondary antibody at 1–200 final dilution (BA-1000, Vector Laboratories). DAB Map including 3,3-Diaminobenzidine chromogen was applied to the slides for histological staining.

Digital images of Masson’s trichrome stained sections were captured at 20X magnification using Aperio AT2 scanner (Aperio Leica) and analyzed with the HALO image analysis platform (Indica labs). A tissue classifier add-on was applied to identify large vessels and myocardial tissue using machine learning with random forest algorithm based on color texture and contextual features. A positive pixel count was applied to quantify the collagen ratio with HALO area quantification algorithm in the interstitial myocardial tissue and large vessels. Microvasculature was quantified on CD31-stained slides. Five regions of interest (2 mm^2^ each) were selected in transversal sectioned myofibers of the left ventricle. Microvessel density (with a vessel area ≤5000 mm^2^) was measured using ImageScope microvessels algorithm (Aperio Leica) and expressed as microvascular density/mm^2^.

### 2.7. Data and statistical analysis

The data and statistical analysis comply with the recommendations on experimental design and analysis in pharmacology [23]. In each study (adult and aged animals groups), statistical analyses were performed at baseline on each parameter to compare Le-ZSF1 rats to the 2 groups of Ob-ZSF1 rats prior to randomization. Then, after 4-weeks of treatment, the pathology in the Ob-ZSF1-CTRL group was evaluated by statistical comparison to the Le-ZSF1-CTRL group. The effect of SAR247799 treatment was then compared to control chow treatment in Ob-ZSF1 rats. For each analysis, when the hypothesis of normality was fulfilled, a Student test was performed, otherwise a Wilcoxon test was performed. p<0.05 was considered significant. The approach to perform distinct analyses before and after treatment was chosen in order to perform the statistical analyses taken into account exactly the same experimental conditions, and avoid impact of these on the results. Moreover, a distinct statistical analysis at baseline allows to take into account all the ZSF1 animals to evaluate the pathology effect with more power. Post treatment two models were performed to answer two distinct objectives: evaluation of the pathology and then of the treatment effect. One model by objective was chosen to gain power in case of heteroscedasticity. Data are expressed as mean ± SEM or median ± IQR (interquartile range). All statistical analyses were performed with SAS® version 9.4 for Windows 7. The authors confirm that the data supporting the findings of this study are available within the article.

## 3. Results

### 3.1. Characteristics of adult and aged obese ZSF1 rats

Compared to age-matched Le-ZSF1 rats, 26-week-old Ob-ZSF1 rats developed slight cardiac hypertrophy (significantly increased left ventricular posterior wall at end-diastole, LVPWd, thickness) and diastolic dysfunction (significantly increased E/e’ ratio), together with a preserved ejection fraction % **(S1 Table)**. 64-week-old Ob-ZSF1 rats, compared to their age-matched Le-ZSF1, displayed diastolic dysfunction (significantly increased E/e’) and significantly more pronounced left ventricular hypertrophy, with both thickened inter-ventricular septum at end-diastole (IVSd) and LVPWd. In both studies, Ob-ZSF1 rats displayed a significantly increased stroke volume and similar cardiac index compared to age-matched Le-ZSF1 rats. Relative to age-matched Le-ZSF1 rats, chambers volumes were preserved in the adult animals, while end-diastolic volume increased significantly in the aged animals.

### 3.2. SAR247799 delays progression of diastolic dysfunction in adult and aged obese ZSF1 rats

**Fig 2:**
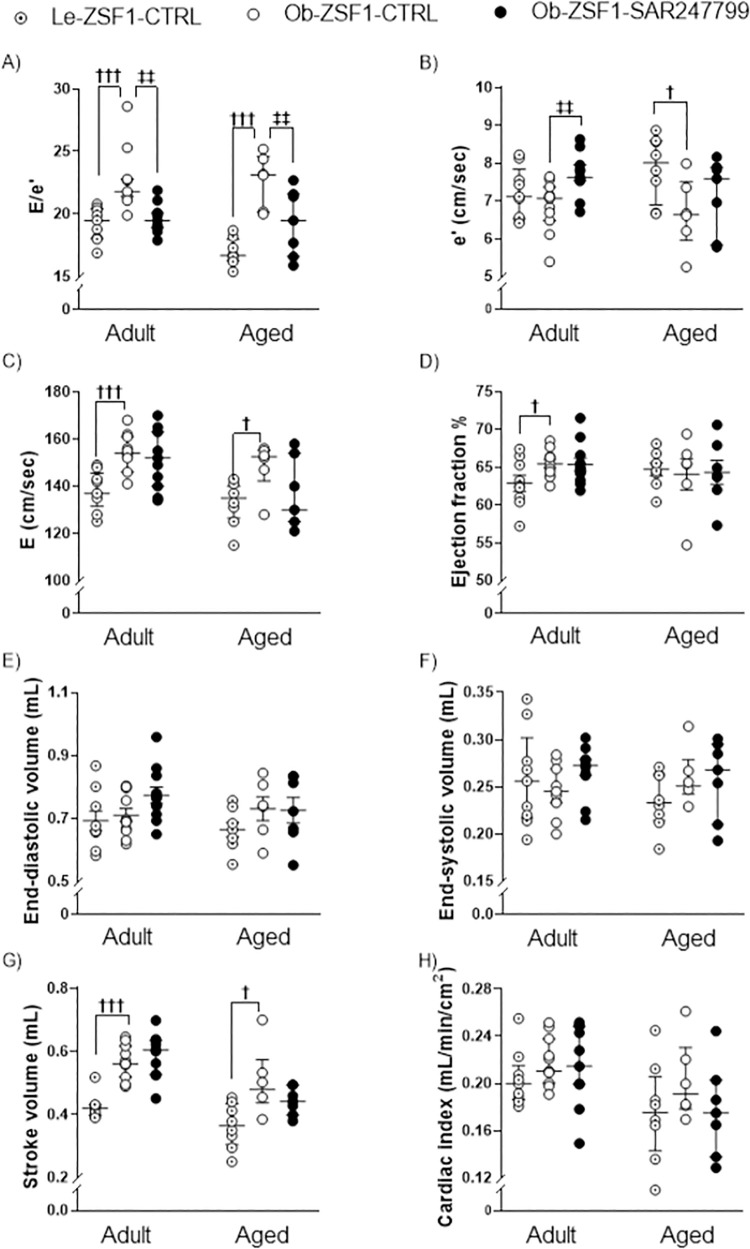
Cardiac diastolic and systolic function in adult and aged ZSF1 rats after 4 weeks of treatment. Diastolic function was evaluated by (A) E/e’ ratio (B) septal peak e’ wave velocity at mitral annulus level and (C) mitral valve peak E wave velocity. Cardiac systolic function was assessed by (D) ejection fraction, (E) end-diastolic volume, (F) end-systolic volume, (G) stroke volume and (H) cardiac index. Data are expressed mean ± SEM, except E, e’, E/e’, end-systolic volume, stroke volume and which are expressed as median ± IQR. **†**p<0.05, **†††** p<0.001: comparison of Le-ZSF1-CTRL to Ob-ZSF1-CTRL using a Student t-test except for E/e’ and stroke volume for adult animals and for E wave for aged animals for which a Wilcoxon test was performed. **‡**p<0.05, **‡‡**p<0.01: p-values obtained from the comparison between Ob-ZSF1-CTRL and Ob-ZSF1-SAR247799 using a Student t-test except for E/e’ for adult animals and for E wave for aged animals for which a Wilcoxon test was performed. For adult animals, Le-ZSF1-CTRL N = 9, Ob-ZSF1-CTRL N = 11 and Ob-ZSF1-SAR247799 N = 11. For aged animals Le-ZSF1-CTRL N = 8–9, Ob-ZSF1-CTRL N = 6–7 and Ob-ZSF1-SAR247799 N = 7; N = number of rats. A lower number of n values is due to technical issue in echocardiography data acquisition.

The effect of SAR47799 on diastolic function was assessed by measuring the E/e’ ratio, an index of ventricular filling pressure, by echocardiography. SAR247799 consistently limited the deterioration of diastolic function, as shown by a significant reduction of the E/e’ ratio **(Fig 2A)** in both adult and aged ZSF1 rats. This reduced E/e’ was mainly due to restored mitral annular velocity during early diastole (e’) and to lesser extent to improved mitral flow velocity during early diastole (E wave) **(Fig 2B and 2C)**. E/A ratio measured in adult animals did not reach statistical significance difference in the model nor with the compound (**S1 Fig**). SAR247799 did not modify indices of systolic function including ejection fraction % **(Fig 2D)**, the end-diastolic volume and the end-systolic volume **(Fig 2E and 2F)**, the stroke volume or the cardiac index **(Fig 2G and 2H)** in either adult or aged ZSF1 rats.

### 3.3. SAR247799 reduced cardiac hypertrophy in aged ZSF1 rats

**Fig 3:**
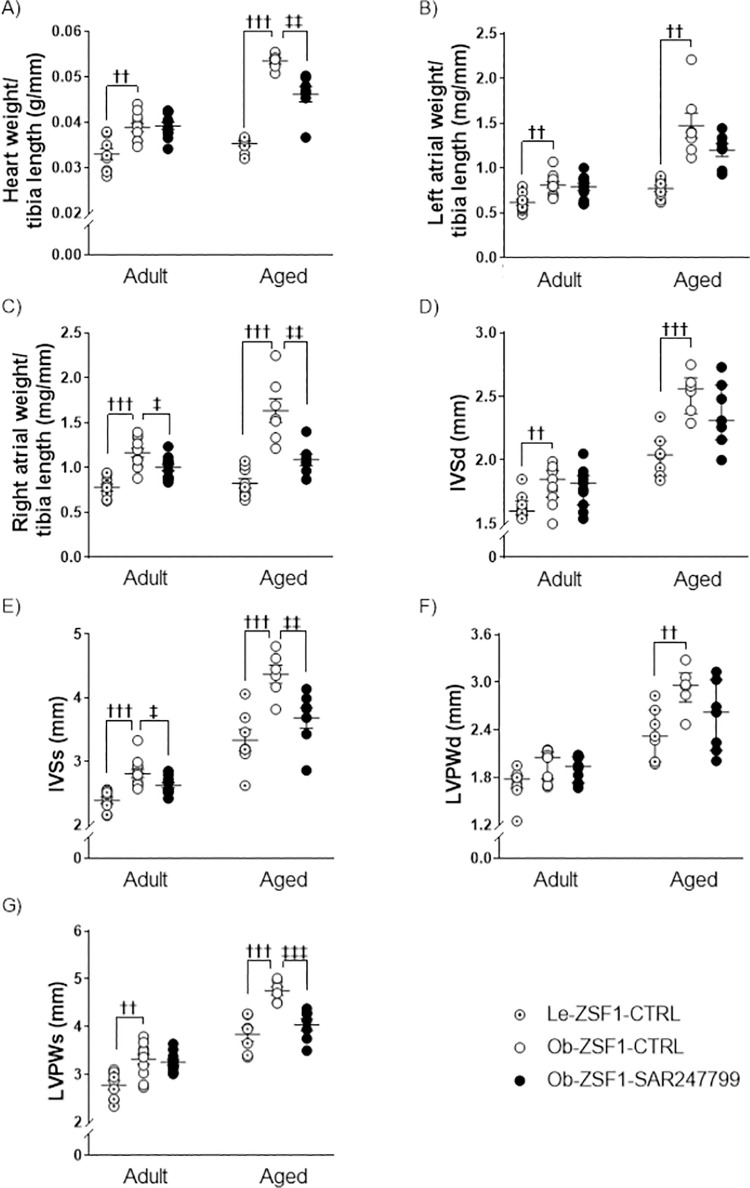
Cardiac hypertrophy in adult and aged ZSF1 rats after 4 weeks of treatment. **(A)** Heart weight/tibia length, **(B)** left atrial weight/tibia length, **(C)** right atrial weight/tibia length, **(D)** interventricular septum thickness at end-diastole (IVSd), **(E)** interventricular septum thickness at end-systole (IVSs), **(F)** left ventricular posterior wall thickness at end-diastole (LVPWd), **(G)** left ventricular posterior wall thickness at end-systole (LVPWs). Data expressed as mean ± SEM, except IVSd and LVPWd which are expressed as median ± IQR. **††** p<0.01, **†††** p<0.001: comparison of Le-ZSF1-CTRL to Ob-ZSF1-CTRL using a Student t-test, except LVPWd (adult animals) for which a Wilcoxon test was performed. **‡**p<0.05, **‡‡**p<0.01, **‡‡‡**p<0.001: comparison between Ob-ZSF1-CTRL and Ob-ZSF1-SAR247799 using a Student t-test, except LVPWd (adult animals) for which a Wilcoxon test was performed. For adult animals, Le-ZSF1-CTRL N = 9, Ob-ZSF1-CTRL N = 11 and Ob-ZSF1-SAR247799 N = 10–11. For aged animals Le-ZSF1-CTRL N = 8–9, Ob-ZSF1-CTRL N = 6–7 and Ob-ZSF1-SAR247799 N = 7; N = number of rats. A lower number of n values is due to technical issue in echocardiography data acquisition.

The degree of cardiac hypertrophy was quantified in the two models by heart and heart chamber weights as well as by chamber morphometric parameters assessed by echocardiography **(Fig 3)**. Compared to age-matched Le-ZSF1-CTRL rats, Ob-ZSF1-CTRL rats displayed increase in total cardiac hypertrophy (evaluated as heart weight/tibia length), left atrial weight/tibia length, right atrial weight/tibia length, inter-ventricular septum at end-diastole (IVSd), inter-ventricular septum at end-systole (IVSs), left ventricular posterior wall at end-diastole (LVPWd) and left ventricular posterior wall at end-systole (LVPWs) **(Fig 3A–3G),** more marked in aged than in adult animals. In aged animals, SAR247799 significantly reduced heart weight/tibia length **(Fig 3A)**, right atrial weight/tibia length **(Fig 3C)**, inter-ventricular septum at end-systole (IVSs) **(Fig 3E)** and LVPWs **(Fig 3G)** and showed a trend to reduce left atrial weight/tibia length **(Fig 3B)**, IVSd **(Fig 3D)** and LVPWd **(Fig 3F)**. In adult animals, SAR247799 significantly reduced right atrial weight/tibia length **(Fig 3C)** and IVSs **(Fig 3E)**, while have little effect on the other parameters. Overall SAR247799 reduced cardiac hypertrophy in both adult and aged animals groups, but the anti-hypertrophic effect was more pronounced in aged animals which exhibited more advanced hypertrophy. Representative echocardiographic images for E wave, e’ wave and left ventricular thickness from TM-mode are shown in **S2(A)–S2(C) Fig** for adult animals and **S2(D)–S2(F) Fig** for aged animals.

### 3.4. SAR247799 reduced cardiac fibrosis in aged ZSF1 rats independent of microvascular density

**Fig 4:**
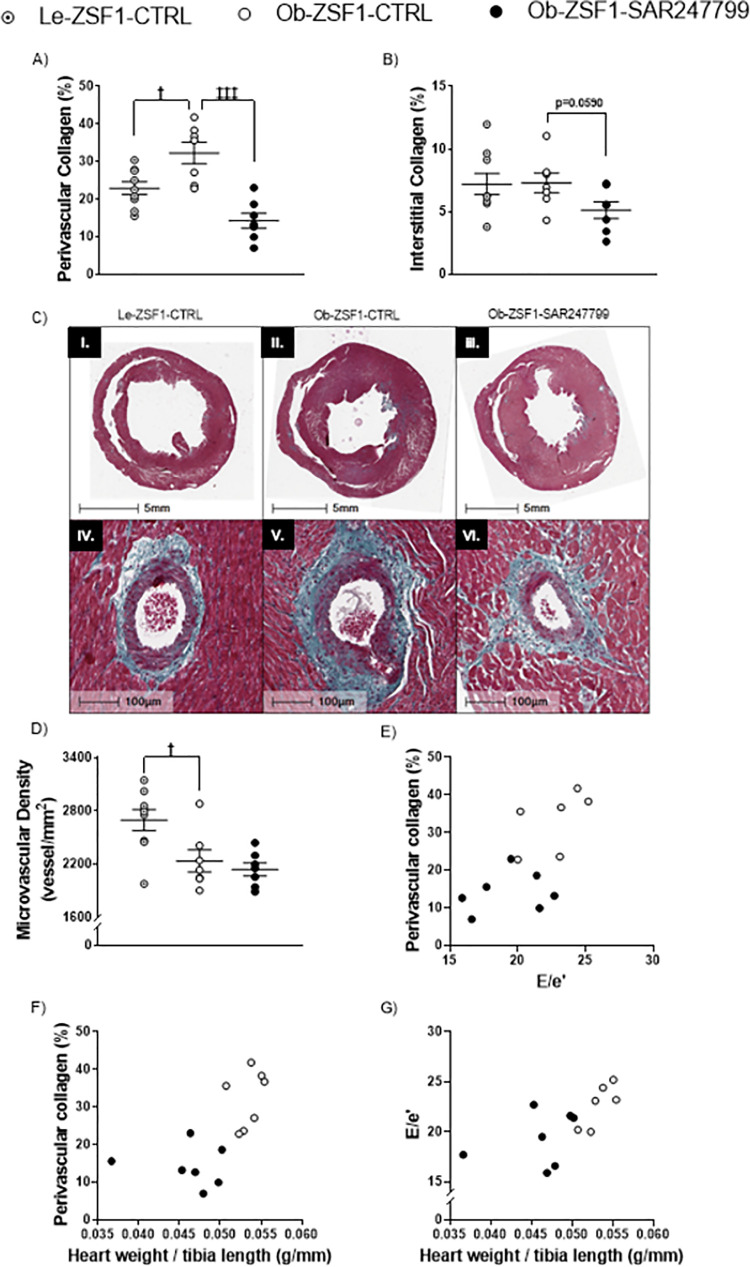
Cardiac fibrosis and microvascular density after 4 weeks of treatment and relationships between diastolic dysfunction, cardiac hypertrophy and fibrosis in aged ZSF1 rats. **(A)** perivascular collagen, **(B)** interstitial collagen, **(C)** representative histological images of cardiac fibrosis in Le-ZSF1-CTRL (I.), Ob-ZSF1-CTRL (II.) andOb-ZSF1-SAR247799 (III.), scale bar 5 mm and corresponding perivascular fibrosis (IV., V. and V.,) stained in green by Masson trichrome staining, scale bar, 100 μm. **(D)** microvascular density. Scatter plot showing relationships between **(E)** perivascular collagen and diastolic dysfunction (E/e’), **(F)** perivascular collagen and heart weight/ tibia length (normalized cardiac hypertrophy), **(G)** diastolic dysfunction (E/e’) and heart weight/ tibia length. For **(A, B and D)** data presented as mean ± SEM. **†**p<0.05: p-values obtained to compare Le-ZSF1-CTRL to Ob-ZSF1-CTRL using a Student t-test. **‡‡‡**p<0.001: p-values obtained from the comparison between Ob-ZSF1-CTRL and Ob-ZSF1-SAR247799 using a Student t-test. Le-ZSF1-CTRL N = 9, Ob-ZSF1-CTRL N = 7 and Ob-ZSF1-SAR247799 N = 7; N = number of rats.

Compared to Le-ZSF1-CTRL rats, Ob-ZSF1-CTRL rats displayed an increase in perivascular collagen in the aged ZSF1 rats and similar levels of interstitial collagen **(Fig 4A–4C)**. SAR247799 significantly reduced perivascular collagen and tended to decrease interstitial collagen. Microvascular density in the left ventricle was decreased in Ob-ZSF1-CTRL rats compared to Le-ZSF1-CTRL. SAR247799 did not restore cardiac microvascular density over the 4-week treatment period **(Fig 4D)**.

### 3.5. SAR247799 concomitantly ameliorated diastolic function, fibrosis and hypertrophy indices in aged ZSF1 rats

Relationships between the effects of SAR247799 on diastolic function, hypertrophy and fibrosis, were illustrated with scatter plots for the respective parameters, E/e’, heart weight/tibia length and perivascular collagen. SAR247799-treated rats displayed clear separated values when compared to Ob-ZSF1-CTRL rats for the relationships involving perivascular collagen with diastolic dysfunction (E/e’) **(Fig 4E)**, perivascular collagen with normalized cardiac hypertrophy (heart weight/tibia length) **(Fig 4F)** and diastolic dysfunction with normalized cardiac hypertrophy **(Fig 4G)**. Overall, SAR247799 improved diastolic function, hypertrophy and fibrosis concomitantly.

### 3.6. SAR247799 improved endothelial function without modifying blood pressure

**Fig 5:**
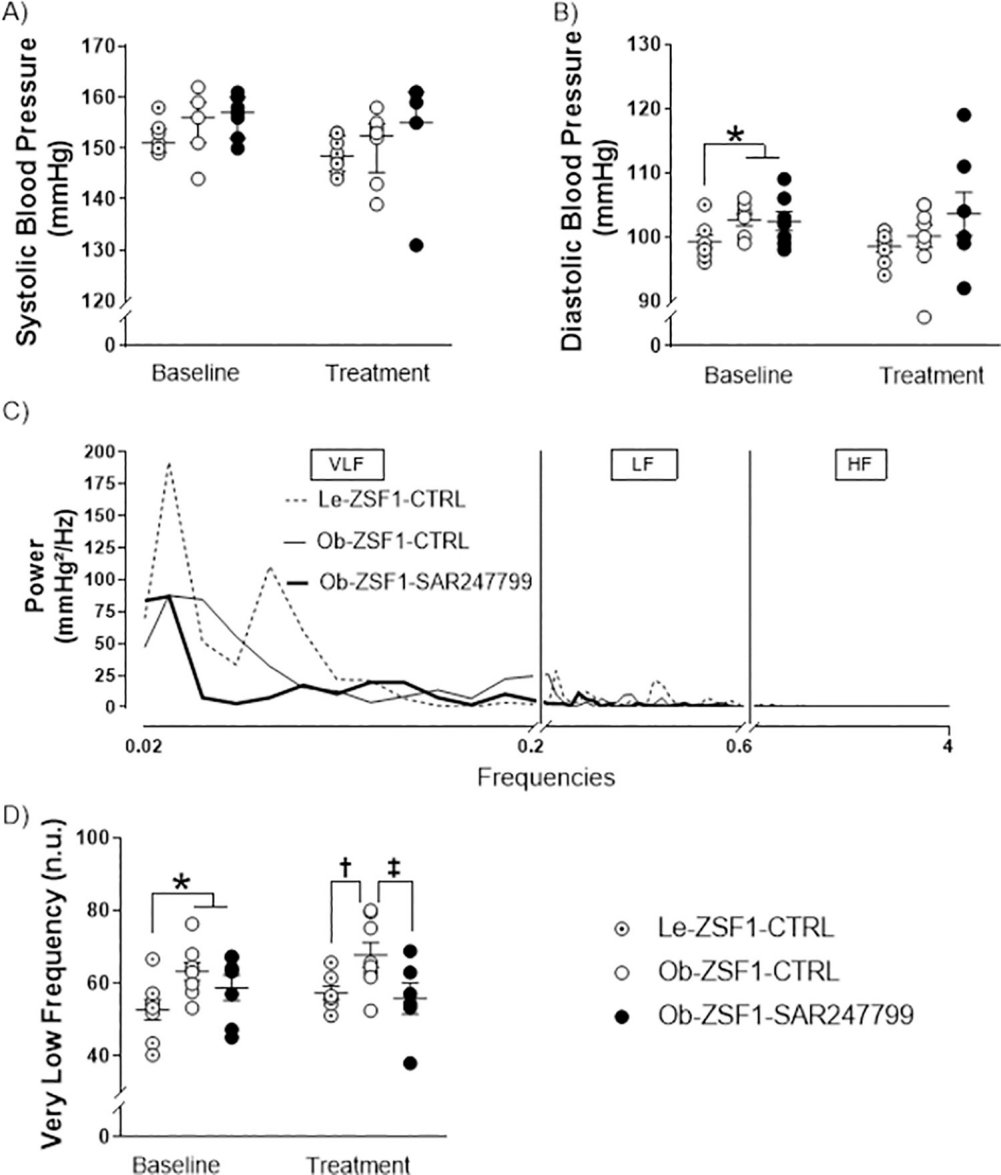
Blood pressure and blood pressure variability after 4 weeks of treatment in adult animals. **A)** systolic blood pressure (SBP), **(B)** diastolic blood pressure, **(C)** representative spectra of systolic blood pressure variability showing very low frequency (VLF), low frequency (LF) and high frequency (HF) bands. **(D)** very low frequency (n.u.) bands from spectral analysis of SBP variability. Data presented as mean ± SEM, except SBP who is presented as median ± IQR. *p<0.05, **p<0.01: p-values obtained to compare Le-ZSF1-CTRL to Ob-ZSF1 (Ob-ZSF1-CTRL and Ob-ZSF1-SAR247799 pooled) using a Student t-test. A Student t-test was also performed to compare Ob-ZSF1-CTRL to Ob-ZSF1-SAR247799 at baseline. No significant difference was observed between the two randomized groups at baseline for any of the parameters. **†**p<0.05: p-values obtained to compare Le-ZSF1-CTRL to Ob-ZSF1-CTRL using a Student t-test. **‡**p<0.05: p-values obtained from the comparison between Ob-ZSF1-CTRL and Ob-ZSF1-SAR247799 using a Student t-test except for SBP for which a Wilcoxon test was performed. For adult animals, Le-ZSF1-CTRL N = 8, Ob-ZSF1-CTRL N = 8 and Ob-ZSF1-SAR247799 N = 7; N = number of rats. The lower number of n values is due to technical issue in telemetry data acquisition.

Vascular effects of SAR247799 were investigated in adult ZSF1 rats, previously implanted with a transducer allowing pressure measurement of the abdominal aorta. At baseline, Ob-ZSF1 rats displayed similar systolic and an increased diastolic blood pressure compared to Le-ZSF1 rats. SAR247799 did not modify systolic or diastolic blood pressure (**Fig 5A and 5B**).

We further investigated systolic blood pressure variability by spectral methods and computed VLF, LF and HF (**Fig 5C**) bands of the power of the spectrum. Frequency analysis of blood pressure tracing using Fourier transformation consist of a quantitative measurement of blood pressure variability at distinctive frequency bandwidths that have been associated with blood pressure control mechanisms. Indeed, vasoactive agents may affect variability without changing absolute blood pressure [22, 24]. In rats, HF bands of SBP variability have been associated with β-adrenoceptor-mediated cardiac sympathetic function or to the mechanical effect of respiration [25], LF bands to vascular sympathetic activity, and VLF bands to endothelial-derived nitric oxide action [22]. Thereby, the integral of VLF (0.02–0.2 Hz) is inversely associated with the activity of the endothelial nitric oxide system [22]. In our study VLF was significantly higher in Ob-ZSF1-CTRL rats compared to Le-ZSF1-CTRL rats and SAR247799 significantly reduced this parameter, implying an improvement in endothelial function **(Fig 5D)**.

### 3.7. SAR247799 improved renal glomerular function in aged ZSF1 rats

Renal function parameters in Ob-ZSF1 and Le-ZSF1 rats in adult and aged ZSF1 rats, at baseline and after 4 weeks of treatment are summarized in **Table 1**. In aged animals, Ob-ZSF1 rats displayed a decrease in urinary function compared to Le-ZSF1 littermates, as demonstrated by significant increases in protein/creatinine ratio, FENa and FECl. Urinary flow was elevated at baseline in Ob-ZSF1-CTRL rats compared to Le-ZSF1, and to a lesser extent 4 weeks later, consistent with the beginning of end-stage renal failure. Four-week treatment with SAR247799 significantly decreased urinary protein/creatinine ratio and FECl, indicating that renal filtration was ameliorated. In adult animals, SAR247799 did not modify renal parameters in Ob-ZSF1 rats that started to display renal dysfunction (increased urinary flow, urinary protein/creatinine ratio and FENa).

**Table 1:** Renal function and physiological parameters at baseline and after 4 weeks of treatment.

		Adult animals			Aged animals		
		Le-ZSF1	Ob-ZSF1-*CTRL*	Ob-ZSF1-*SAR247799*	Le-ZSF1	Ob-ZSF1-*CTRL*	Ob-ZSF1-*SAR247799*
		(n = 9)	(n = 9–11)	(n = 11)	(n = 8–9)	(n = 6–7)	(n = 6–7)
**BASELINE**	Urinary Flow (μL/min)	5.34 ± 0.678***	36.7 ± 5.92	36.7 ± 7.01	8.08 ± 0.860***	34.5 ± 2.44	32.3 ± 1.17
	Protein/Creatinine Ratio (g/μmol)	0.095 ± 0.006***	2.06 ± 0.110	2.49 ± 0.193	0.133 ± 0.009***	5.80 ± 0.491	5.52 ± 0.482
	Glycosuria (mmol)/ 24h	0.032 ± 0.004 ***	38.2 ± 6.64	36.8 ± 8.75	0.055 ± 0.005 *	0.132 ± 0.051	0.133 ± 0.051
	Creatinin Clearance (μL/min)	3530 ± 306 **	5163 ± 405	4935 ± 322	2815 ± 158 ***	1465 ± 157	1556 ± 182
	FENa	0.303 ± 0.031	0.338 ± 0.026	0.366 ± 0.043	0.294 ± 0.020***	0.898 ± 0.136	0.765 ± 0.089
	FECl	1.19 ± 0.238***	0.854 ± 0.251	0.863 ± 0.365	0.807 ± 0.205***	1.69 ± 1.03	1.53 ± 0.924
	Body weight (g)	493 ± 24***	616 ± 61	610 ± 109	619 ± 45**	887 ± 21.8	852 ± 63
	Fructosamine (μmol/L)	162 ± 2***	204 ± 6	208 ± 6	188 ± 3	184 ± 6	185 ± 10
	Lymphocyte number (10^3^/μL)	6.61 ± 0.450	7.10 ± 0.353	7.98 ± 0.520	6.77 ± 0.654	7.87 ± 0.749	7.60 ± 0.548
	Heart Rate (bpm)	312 ± 34.5*	286 ± 20.5	288 ± 25.5	318 ± 62*	300 ± 26	289 ± 33
		**Adult animals**			**Aged animals**		
		**Le-ZSF1-CTRL**	**Ob-ZSF1-CTRL**	**Ob-ZSF1-SAR247799**	**Le-ZSF1-CTRL**	**Ob-ZSF1-CTRL**	**Ob-ZSF1-SAR247799**
		**(n = 9)**	**(n = 9–11)**	**(n = 11)**	**(n = 8–9)**	**(n = 6–7)**	**(n = 6–7)**
**ON-TREATMENT**	Urinary Flow (μL/min)	6.22 ± 0.524	36.8 ± 3.86^†††^	34.1 ± 2.82	15.1 ± 2.64	26.9 ± 5.58	26.1 ± 4.30
	Protein/Creatinine Ratio(g/μmol)	0.091 ± 0.007	2.82 ± 0.177^†††^	3.18 ± 0.196	0.294 ± 0.029	10.3 ± 0.621 ^†††^	8.17 ± 0.231^‡^
	Glycosuria (mmol)/ 24h	0.018 ± 0.002	18.9 ± 3.07 ^**†††**^	17.4 ± 2.8	0.011 ± 0.002	0.022 ± 0.008	0.021 ± 0.008
	Creatinin Clearance (μL/min)	2599 ± 190	3819 ± 369 ^**†**^	3451 ± 519	1428 ± 330	351 ± 78	465 ± 76
	FENa	0.258 ± 0.017	0.395 ± 0.045^**†**^	0.401 ± 0.040	0.518 ± 0.073	1.32 ± 0.072 ^**†††**^	1.10 ± 0.092
	FECl	1.32 ± 0.119	1.28 ± 0.637	1.28 ± 0.648	1.19 ± 0.783	4.16 ± 0.669 ^**†††**^	2.63 ± 0.898^**‡‡**^
	Plasma SAR247799 (ng/mL)	-	-	3627 ± 394	-	-	504 ± 125
	Body weight (g)	529 ± 60	654 ± 122^†††^	681 ± 134	601 ± 45.5	774 ± 42^†††^	731 ± 86
	Fructosamine (μmol/L)	160 ± 1	201 ± 6^†††^	209 ± 5	191 ± 4	186 ± 5	189 ± 7
	Lymphocyte number (10^3^/μL)	6.43 ± 0.202	7.07 ± 0.231	6.11 ± 0.211^‡‡^	4.00 ± 0.422	5.25 ± 0.822	7.09 ± 1.15
	Heart Rate (bpm)	307 ± 35.1	291 ± 24	279 ± 13	340 ± 29	331 ± 18.2	312 ± 28^**‡**^

FENa, fractional excretion of sodium; FECl, fractional excretion of chloride.

Data are presented as mean ± SEM, except for FECl, body weight and heart rate which are represented as median ± IQR

For baseline evaluation

*p<0.05

**p<0.01

***p<0.001: p-values obtained to compare Le-ZSF1 to Ob-ZSF1 (Ob-ZSF1-CTRL and Ob-ZSF1-SAR247799 pooled) using a Student t-test except for FECl, body weight and heart rate for adult animals and body weight for aged animals for which a Wilcoxon test was performed. A Student t-test was also performed to compare Ob-ZSF1-CTRL to Ob-ZSF1-SAR247799 at baseline. No significant difference was observed between the two Ob-ZSF1 groups at baseline for any of the parameters.

For on-treatment evaluation, for adult and aged animals with HFpEF: **†**p<0.05, **††**p<0.01, **†††**p<0.001: p-values obtained from the comparison between Le-ZSF1-CTRL and Ob-ZSF1-CTRL using a Student t-test for all parameters.

**‡**p<0.05, **‡‡**p<0.01: p-values obtained from the comparison between Ob-ZSF1-CTRL and Ob-ZSF1-SAR247799 using a Student t-test except for heart rate for adult animals for which a Wilcoxon test was performed. A lower number of n values is due to technical issue in sample data collection or analysis.

### 3.8. Effects of SAR247799 on physiological parameters

The effect of SAR247799 on lymphocytes (a marker of S1P_1_ desensitization) and heart rate (a marker of cardiac S1P_1_ activation) were determined following 4-week treatment, alongside measures of body weight and compound exposure (**Table 1**). Following the 4-week treatment periods in adult and aged animals, blood lymphocyte counts were similar between Ob-ZSF1-CTRL and Ob-ZSF1-SAR247799 groups, indicating that the dosing regimens did not cause S1P_1_ desensitization. Moreover, SAR247799 reduced heart rate by 4% (NS) and 6% (significant), in adult and aged ZSF1 rats, respectively, compared to the Ob-ZSF1-CTRL group. Ob-ZSF1 rats (both models) were obese compared to age-matched Le-ZSF1 rats, and SAR247799 treatment did not modify body weight. Plasma fructosamine was increased in Ob-ZSF1 rats compared to Le-ZSF1 in adult but not in aged animals and these levels were not affected by SAR247799 treatment (**Table 1**).

Daily food intake at two weeks after treatment in adult and aged rats are shown in **S2A Table**. SAR247799 was incorporated in the chow at the dose of 0.025% (w/w) as a previous study from our laboratory demonstrated the effect of this dose in endothelial protection in another rat model of metabolic dysfunction [17]. In this study, the plasma concentration of SAR247799 after 4 weeks of treatment was 7-fold higher in adult compared to the aged ZSF1 rats. However, daily food intake was greater in adult than in aged animals (**S2B Table**).

## 4. Discussion

The present study provides the first evidence that SAR247799 confers cardio-protective effects in experimental models of LVH and diastolic dysfunction, due to S1P_1_ activation. We demonstrated that 4-week treatment with the G-protein-biased S1P_1_ agonist, SAR247799, blunted the progression of diastolic dysfunction, and reduced cardiac hypertrophy, right atrial enlargement and perivascular cardiac fibrosis. SAR247799 also displayed some renal-protective effects and did not modify arterial blood pressure. The protective effects of SAR247799 were under conditions where there was no S1P_1_ desensitization (no lymphocyte reduction) and there was an improvement in VLF as an endothelial function marker, in line with the proposed mechanism of action.

The Ob-ZSF1 rat mimicked closely the features found in HFpEF patients, i.e. LVH and diastolic dysfunction. HFpEF patients are characterized by having a preserved ejection fraction and impaired diastolic function, as was seen in the Ob-ZSF1 rats [19, 20]. Hypertension, obesity and diabetes are significant human risk factors affecting the progression to HFpEF and were characteristic features of Ob-ZSF1 rats. Old age is an important risk factor for the LVH and diastolic dysfunction progression. The inclusion of the aged Ob-ZSF1 cohort (65 weeks of age) revealed higher degrees of concentric cardiac hypertrophy and atrial remodeling compared to the adult Ob-ZSF1 cohort (31 weeks of age), features described in more than 50% of HFpEF patients [6]. Adult ZSF1 displayed diastolic dysfunction with minimal left ventricular hypertrophy while in aged ZSF1 left ventricular hypertrophy was highly present with diastolic dysfunction. This evidence suggested that left ventricular hypertrophy is establishing with ageing and consequently to diastolic dysfunction and uncontrolled blood pressure. Myocardial fibrosis usually presents as perivascular and fine interstitial reactive fibrosis and is more evident in advanced HFpEF [20, 26], thus was here evaluated in aged ZSF1 rats. In patients, myocardial fibrosis is associated with death and hospitalization for heart failure in proportion to the severity of fibrosis [26]. In our aged Ob-ZSF1, a good amount of perivascular but not interstitial fibrosis was found. Fibrotic tissue deposition in perivascular spaces impairs oxygen supply to the cardiomyocyte. Indeed, perivascular fibrosis is inversely correlated with coronary flow reserve in heart failure patients [27]. Importantly, our results showed that SAR247799 displayed cardio-protective effects in both adult and aged ZSF1 rats, suggesting its potential utility in a broad range of HFpEF patients.

The protective effects of SAR247799 in Ob-ZSF1 rats could be attributed to its S1P_1_-mediated endothelial-protective properties. Previous studies demonstrate that S1P_1_ activation improves outcomes after myocardial or cerebral ischemia/reperfusion injury [28] and that cardiac overexpression of S1P_1_ improves left ventricular contractility and relaxation in rats undergoing myocardial infarction [14], while endothelial S1P_1_ deficient mice display worse outcomes [29]. Indeed, we showed in the present study in Ob-ZSF1 rats, that SAR247799, through its mechanism of action on endothelial function, reduced VLF bands of systolic blood pressure variability, an index of endothelial nitric oxide pathway activation [24]. Similarly, our previous work shows that SAR247799 improves coronary microvascular reserve in a pig model of ischemic injury [16] and endothelial and renal function in diabetic rats [17]. Furthermore, SAR247799 improves flow-mediated dilation in type-2 diabetes patients [17].

As active relaxation is an energy-dependent phase of diastole, vulnerable to ischemia, SAR247799’s ability to improve coronary microvascular perfusion [16] could explain the improvements in diastolic function. Endothelial cells also have an important role in paracrine signaling to the cardiomyocyte through cyclic guanosine monophosphate activation and regulating titin hyperphosphorylation and subsequent cardiac hypertrophy [20]. Given the improvements in hypertrophy seen in the model, the anti-hypertrophic effects of SAR247799 could have been mediated by restoring this paracrine signaling.

The anti-hypertropic and anti-fibrotic effects in the heart shown herein with SAR247799 are consistent with a recent study in which endothelial-specific S1P1 knockdown promoted cardiac hypertrophy and fibrosis in a pressure overload model [30]. Furthermore, the protective effects seen with SAR247799 in the aged ZSF1 rats (improved diastolic function, reduced cardiac hypertrophy, reduced cardiac fibrosis and improved endothelial and renal function) were at plasma concentrations (mean+SD 504+125 ng/ml) that were in a similar range to concentrations causing endothelial-protective effects in humans, rats and pigs, as well as on endothelial cells (see **Table 2**). There is evidence for microvascular rarefaction in HFpEF patients, as coronary microvascular density is reduced in cardiac autopsies [8, 31]. Similarly, our experimental ZSF1 rat model had reduced microvascular density in aged animals. Although S1P_1_ activation plays an essential role in angiogenesis and vasculogenesis, 4-week treatment with SAR247799 did not improve microvascular density. Consequently, the significant improvements in cardiac parameters found with SAR247799, over this timeframe, cannot be explained by changes in microvessel density, but may be more attributed to structural and functional modifications of the vessels. As 12-week S1P_1_ gene therapy in ischemic rats has been shown to improve coronary microvessel density [14], the potential for higher efficacy with longer treatment durations of SAR247799 warrants consideration.

**Table 2:** Comparison of the protective effects of SAR247799 in aged obese ZSF1 rats with effects seen in prior experimental systems.

Experimental system	Protective effects shown	Plasma concentration associated with effects (ng/ml)	Reference
		Mean±SD	
Aged Ob-ZSF1 rats	Improved diastolic function	504±125	Current study
	Reduced cardiac hypertrophy		
	Reduced cardiac fibrosis		
	Improved endothelial function (VLF)		
	Reduced urinary protein/creatinine ratio		
	Reduced fractional excretion of electrolytes		
ZDF rat (intermediate dose strength)	Reduced plasma endothelial biomarkers (sICAM1, sE-Selectin, VWF, nitrite/nitrate)	423±172	[16]
	Reduced urinary protein/creatinine ratio		
	Reduced fractional excretion of electrolytes		
Type-2 diabetes patients (5 mg/day)	Improved endothelial function measured by brachial flow-mediated dilation	779±186	[16]
Rat acute kidney injury model (1 to 3 mg/Kg)^a^	Reduced serum creatinine and urea	867±295 to 2600±884	[15]
	Reduced interstitial hemorrhage		
	Reduced albumin leakage into renal tissue		
	Improved renal PECAM-1 staining		
Pig coronary ischemia reperfusion (1 mg/kg)	Improved vascular reactivity of microcirculation (coronary flow reserve)	2080±166	[15]
Endothelial cells	Barrier integrity (impedance)	EC_50_ = 592	[15]
	Phosphorylation of ERK and AKT	EC_50_ = 171–277	

^a^ 1 mg/kg concentrations calculated by linear extrapolation from 3 mg/kg.

S1P_1_ is also expressed directly on the cardiomyocyte and S1P_1_ activation causes heart rate reduction [9]. In principle, a reduction in heart rate could be associated with cardio-protective effects, especially as cardiac overexpression of S1P_1_ has been shown to improve outcomes after experimentally induced myocardial infarction or stroke [28]. However, SAR247799 has a low volume of distribution in rats and humans and this property limits concentrations in the heart relative to the vasculature [16]. Indeed, SAR247799 has endothelial-protective effects in type-2 diabetes patients under conditions where there were only small changes in heart rate [17]. Similarly, in the current studies SAR247799 caused 4 and 6% reductions in heart rate in the adult- and aged ZSF1 rats, respectively. These levels of heart rate reduction are unlikely to explain the cardioprotective effects seen in the present work. In addition, heart rate-lowering drugs have not demonstrated benefit in patients with HFpEF [32], lending support for a more endothelial-mediated mechanism.

Chronic kidney disease is prevalent in HFpEF patients and is associated with increased morbidity and mortality [33]. Ob-ZSF1 rats inherently develop renal disease and model the cardiorenal syndrome of HFpEF patients [34]. In the present study, Ob-ZSF1 rats developed age-dependent polyuria and proteinuria. There was also an increase in the FENa and FECl, in aged ZSF1 rats. Inappropriate urinary Na^+^ and Cl^-^ loss indicates renal salt wasting which is common in CKD and hypertensive patients [35], and SAR247799 tended to decrease such losses in Ob-ZSF1 rats. Such action of SAR247799 could partially restore glomerular-tubular balance, i.e. reducing salt delivery to the proximal tubules and subsequent reabsorption through improving the functional capacity per nephron [35]. Indeed, SAR247799 improves endothelial function at the glomerular level and reduces the severity of acute kidney injury in rats [16], suggesting that it could have acted by improving renal tubules and their re-absorptive mechanisms in the present study.

Importantly, SAR247799 improved diastolic function independent of any blood pressure modulation [36, 37], a feature that distinguishes it from previous molecules tested in Ob-ZSF1 rats and from renin-angiontensin-aldosterone pathway modulators that have failed to show benefit in HFpEF patients [38]. SAR247799 displays here a unique profile of beneficial effects in adult and aged Ob-ZSF1 rats, without affecting blood pressure or obesity. SAR247799 is not a blood pressure lowering agent as it did not reduce hypertension in treated ZSF1 rats in the present study and it did not modify blood pressure in diabetic patients in a previous study [17]. Similarly, SAR247799 cannot be classified as an anti-diabetic drug as in this study and in the previous study in ZDF rats and in diabetic patients [17], it did not lower hyperglycemia. SAR247799 is also the first agent to show diastolic function improvements in Ob-ZSF1 rats by echocardiographic assessment (E/e’). E/e’ is a reliable non-invasive diastolic index translatable to clinical practice compared to invasive methods including tau and left ventricular end-diastolic pressure, as used in other molecules evaluated in Ob-ZSF1 rats [39]. E/e’ is generally assumed to be less sensitive to preload than other echocardiographic indices of diastolic dysfunction such as E/A and therefore yields more accurate estimations of filling pressures [40]. Agents aiming to improve the physiology of endothelium in this complex microvascular pathology remains one of the most promising approach because of the capability of endothelial-driven molecular pathways to reduce myocardial fibrosis, stiffness and dysfunction [8]. Importantly, this is the first time that S1P1 activation has been pharmacologically associated with benefits in a model of LVH and diastolic dysfunction. Thus, SAR247799 is a promising therapeutic approach and deserves to be tested in the clinical context of LVH and diastolic dysfunction, major components of HFpEF.

Nevertheless, the present study had several limitations. 1) Despite adopting the same dosing regimen, steady state plasma concentrations of SAR247799 were 7-faged lower in the aged cohort compared to the adult cohort. We could speculate that age of the animals could have affected absorption, metabolism or excretion parameters. However, plasma exposure in aged animals were still in a similar range to concentrations in our previous published study in ZDF rats where we demonstrated endothelial protection and were not associated with significant lymphocyte reduction, a marker of S1P_1_ desensitization [17]. In both cohorts, heart rate reductions were minimal (4–6%) and there was no evidence for S1P_1_ desensitization (i.e. no significant lymphocyte reduction) following 4-week treatment. Although the more impressive effects of SAR247799 were achieved in the aged cohort, it is still possible that sub-optimal concentrations could have produced sub-optimal efficacy. 2) Four-week treatment duration may not have been sufficient to see changes in microvessel density, and a longer duration study may have been warranted to see effects through this mechanism. 3) For ethical reasons, we did not study mortality in the rat study which is a key outcome in clinical trials in HFpEF. 4) We did not evaluate the cardiac phenotype and the effect of SAR247799 in female rats. This represents a limitation of the study as the prevalence of HFpEF is higher in women than in men. 5) E/A analysis by doppler echocardiography was not feasible in aged ZSF1 rats because of the high heart rate that lead to E and A wave fusions. Speckle tracking echocardiography for the evaluation of left ventricle and atrial strain was not performed in this study. 6) Despite invasive in both animals and human, the hemodynamic evaluation of left ventricular end-diastolic pressure remains the gold standard of HFpEF and was not here assessed. 7) Finally, anti-hypertrophic effect and internal pathway of S1P_1_ activation through SAR247799 were not addressed in this study.

## Supporting information

S1 Fig **(A)** E/A, **(B)** A wave at mitral valve. Parameters were measured in adult animals. In aged animal data were not determined (nd). Data are expressed as mean ± SEM, †p<0.05, comparison of Le-ZSF1-CTRL to Ob-ZSF1-CTRL using a Student t-test. Le-ZSF1-CTRL N = 9, Ob-ZSF1-CTRL N = 11 and Ob-ZSF1-SAR247799 N = 11.
(TIF)

S2 Fig Representative echocardiographic images of E wave, e’ wave and left ventricular thickness in (A), (B) and (C) for adult animals and in (D), (E) and (F) for aged animals, respectively.
(TIF)

S1 Table Baseline cardiac parameters in adult and aged ZSF1 rats. (DOCX)

S2 Table Daily food intake and SAR247799 plasma concentration. A) Daily food intake at two weeks after treatment in adult and aged rats; B) Individual value of daily food intake and SAR247799 plasma concentration.
(DOCX)

## References

[1] Borlaug BA, Paulus WJ. Heart failure with preserved ejection fraction: pathophysiology, diagnosis, and treatment. Eur Heart J. 2011;32(6):670–9. Epub 2010/12/09. doi: 10.1093/eurheartj/ehq426 ; PubMed Central PMCID: PMC3056204. 21138935 PMC3056204

[2] Shah KS, Xu H, Matsouaka RA, Bhatt DL, Heidenreich PA, Hernandez AF, et al. Heart Failure With Preserved, Borderline, and Reduced Ejection Fraction: 5-Year Outcomes. J Am Coll Cardiol. 2017;70(20):2476–86. Epub 2017/11/17. doi: 10.1016/j.jacc.2017.08.074 . 29141781

[3] Silverman DN, Shah SJ. Treatment of Heart Failure With Preserved Ejection Fraction (HFpEF): the Phenotype-Guided Approach. Curr Treat Options Cardiovasc Med. 2019;21(4):20. Epub 2019/04/15. doi: 10.1007/s11936-019-0709-4 . 30982123

[4] Solomon SD, McMurray JJV, Anand IS, Ge J, Lam CSP, Maggioni AP, et al. Angiotensin-Neprilysin Inhibition in Heart Failure with Preserved Ejection Fraction. N Engl J Med. 2019;381(17):1609–20. Epub 2019/09/03. doi: 10.1056/NEJMoa1908655 . 31475794

[5] Anker SD, Butler J, Filippatos G, Ferreira JP, Bocchi E, Bohm M, et al. Empagliflozin in Heart Failure with a Preserved Ejection Fraction. N Engl J Med. 2021;385(16):1451–61. Epub 2021/08/28. doi: 10.1056/NEJMoa2107038 . 34449189

[6] Pfeffer MA, Shah AM, Borlaug BA. Heart Failure With Preserved Ejection Fraction In Perspective. Circ Res. 2019;124(11):1598–617. Epub 2019/05/24. doi: 10.1161/CIRCRESAHA.119.313572 ; PubMed Central PMCID: PMC6534165. 31120821 PMC6534165

[7] Akiyama E, Sugiyama S, Matsuzawa Y, Konishi M, Suzuki H, Nozaki T, et al. Incremental prognostic significance of peripheral endothelial dysfunction in patients with heart failure with normal left ventricular ejection fraction. J Am Coll Cardiol. 2012;60(18):1778–86. Epub 2012/10/09. doi: 10.1016/j.jacc.2012.07.036 . 23040568

[8] Paulus WJ, Tschope C. A novel paradigm for heart failure with preserved ejection fraction: comorbidities drive myocardial dysfunction and remodeling through coronary microvascular endothelial inflammation. J Am Coll Cardiol. 2013;62(4):263–71. Epub 2013/05/21. doi: 10.1016/j.jacc.2013.02.092 . 23684677

[9] Li N, Zhang F. Implication of sphingosin-1-phosphate in cardiovascular regulation. Front Biosci (Landmark Ed). 2016;21:1296–313. Epub 2016/04/23. doi: 10.2741/4458 ; PubMed Central PMCID: PMC4865951. 27100508 PMC4865951

[10] Cartier A, Hla T. Sphingosine 1-phosphate: Lipid signaling in pathology and therapy. Science. 2019;366(6463). Epub 2019/10/19. doi: 10.1126/science.aar5551 . 31624181 PMC7661103

[11] Galvani S, Sanson M, Blaho VA, Swendeman SL, Obinata H, Conger H, et al. HDL-bound sphingosine 1-phosphate acts as a biased agonist for the endothelial cell receptor S1P1 to limit vascular inflammation. Sci Signal. 2015;8(389):ra79. Epub 2015/08/14. doi: 10.1126/scisignal.aaa2581 ; PubMed Central PMCID: PMC4768813. 26268607 PMC4768813

[12] Polzin A, Piayda K, Keul P, Dannenberg L, Mohring A, Graler M, et al. Plasma sphingosine-1-phosphate concentrations are associated with systolic heart failure in patients with ischemic heart disease. J Mol Cell Cardiol. 2017;110:35–7. Epub 2017/07/16. doi: 10.1016/j.yjmcc.2017.07.004 . 28709768

[13] Vaisar T, Couzens E, Hwang A, Russell M, Barlow CE, DeFina LF, et al. Type 2 diabetes is associated with loss of HDL endothelium protective functions. PLoS One. 2018;13(3):e0192616. Epub 2018/03/16. doi: 10.1371/journal.pone.0192616 ; PubMed Central PMCID: PMC5854245. 29543843 PMC5854245

[14] Cannavo A, Rengo G, Liccardo D, Pagano G, Zincarelli C, De Angelis MC, et al. beta1-adrenergic receptor and sphingosine-1-phosphate receptor 1 (S1PR1) reciprocal downregulation influences cardiac hypertrophic response and progression to heart failure: protective role of S1PR1 cardiac gene therapy. Circulation. 2013;128(15):1612–22. Epub 2013/08/24. doi: 10.1161/CIRCULATIONAHA.113.002659 ; PubMed Central PMCID: PMC3952877. 23969695 PMC3952877

[15] Grailhe P, Boutarfa-Madec A, Beauverger P, Janiak P, Parkar AA. A label-free impedance assay in endothelial cells differentiates the activation and desensitization properties of clinical S1P1 agonists. FEBS Open Bio. 2020;10(10):2010–20. Epub 2020/08/19. doi: 10.1002/2211-5463.12951 ; PubMed Central PMCID: PMC7530392. 32810927 PMC7530392

[16] Poirier B, Briand V, Kadereit D, Schafer M, Wohlfart P, Philippo MC, et al. A G protein-biased S1P1 agonist, SAR247799, protects endothelial cells without affecting lymphocyte numbers. Sci Signal. 2020;13(634). Epub 2020/06/04. doi: 10.1126/scisignal.aax8050 . 32487716

[17] Bergougnan L, Andersen G, Plum-Morschel L, Evaristi MF, Poirier B, Tardat A, et al. Endothelial-protective effects of a G-protein-biased sphingosine-1 phosphate receptor-1 agonist, SAR247799, in type-2 diabetes rats and a randomized placebo-controlled patient trial. Br J Clin Pharmacol. 2020; Epub 2020/10/31. doi: 10.1111/bcp.14632 . 33125753 PMC8247405

[18] Bergougnan L, Armani S, Golor G, Tardat A, Vitse O, Hurbin F, et al. First-in-human study of the safety, tolerability, pharmacokinetics and pharmacodynamics of single and multiple oral doses of SAR247799, a selective G-protein-biased Sphingosine-1 phosphate receptor-1 agonist for endothelial protection. Br J Clin Pharmacol. 2020; doi: 10.1111/bcp.14422 Epub 2020/06/11. 32520410 PMC9328431

[19] Schauer A, Draskowski R, Jannasch A, Kirchhoff V, Goto K, Männel A, et al. ZSF1 rat as animal model for HFpEF: Development of reduced diastolic function and skeletal muscle dysfunction. ESC Heart Failure. 2020;7(5):2123–34. doi: 10.1002/ehf2.12915 32710530 PMC7524062

[20] Hamdani N, Franssen C, Lourenco A, Falcao-Pires I, Fontoura D, Leite S, et al. Myocardial titin hypophosphorylation importantly contributes to heart failure with preserved ejection fraction in a rat metabolic risk model. Circ Heart Fail. 2013;6(6):1239–49. Epub 2013/09/10. doi: 10.1161/CIRCHEARTFAILURE.113.000539 . 24014826

[21] Valero-Munoz M, Backman W, Sam F. Murine Models of Heart Failure with Preserved Ejection Fraction: a "Fishing Expedition". JACC Basic Transl Sci. 2017;2(6):770–89. Epub 2018/01/16. doi: 10.1016/j.jacbts.2017.07.013 ; PubMed Central PMCID: PMC5764178. 29333506 PMC5764178

[22] Stauss HM. Identification of blood pressure control mechanisms by power spectral analysis. Clin Exp Pharmacol Physiol. 2007;34(4):362–8. Epub 2007/02/28. doi: 10.1111/j.1440-1681.2007.04588.x . 17324151

[23] Curtis MJ, Alexander S, Cirino G, Docherty JR, George CH, Giembycz MA, et al. Experimental design and analysis and their reporting II: updated and simplified guidance for authors and peer reviewers. British Journal of Pharmacology. 2018;175(7):987–93. doi: 10.1111/bph.14153 29520785 PMC5843711

[24] Desjardins F, Lobysheva I, Pelat M, Gallez B, Feron O, Dessy C, et al. Control of blood pressure variability in caveolin-1-deficient mice: role of nitric oxide identified in vivo through spectral analysis. Cardiovasc Res. 2008;79(3):527–36. Epub 2008/03/20. doi: 10.1093/cvr/cvn080 . 18349137

[25] Yoshimoto T, Eguchi K, Sakurai H, Ohmichi Y, Hashimoto T, Ohmichi M, et al. Frequency components of systolic blood pressure variability reflect vasomotor and cardiac sympathetic functions in conscious rats. J Physiol Sci. 2011;61(5):373–83. Epub 2011/06/30. doi: 10.1007/s12576-011-0158-7 ; PubMed Central PMCID: PMC3168447. 21713646 PMC3168447

[26] de Boer RA, De Keulenaer G, Bauersachs J, Brutsaert D, Cleland JG, Diez J, et al. Towards better definition, quantification and treatment of fibrosis in heart failure. A scientific roadmap by the Committee of Translational Research of the Heart Failure Association (HFA) of the European Society of Cardiology. Eur J Heart Fail. 2019;21(3):272–85. Epub 2019/02/05. doi: 10.1002/ejhf.1406 ; PubMed Central PMCID: PMC6607480. 30714667 PMC6607480

[27] Dai Z, Aoki T, Fukumoto Y, Shimokawa H. Coronary perivascular fibrosis is associated with impairment of coronary blood flow in patients with non-ischemic heart failure. J Cardiol. 2012;60(5):416–21. Epub 2012/08/08. doi: 10.1016/j.jjcc.2012.06.009 . 22867802

[28] Swendeman SL, Xiong Y, Cantalupo A, Yuan H, Burg N, Hisano Y, et al. An engineered S1P chaperone attenuates hypertension and ischemic injury. Sci Signal. 2017;10(492). Epub 2017/08/16. doi: 10.1126/scisignal.aal2722 ; PubMed Central PMCID: PMC5680089. 28811382 PMC5680089

[29] Perry HM, Huang L, Ye H, Liu C, Sung SJ, Lynch KR, et al. Endothelial Sphingosine 1Phosphate Receptor1 Mediates Protection and Recovery from Acute Kidney Injury. J Am Soc Nephrol. 2016;27(11):3383–93. Epub 2016/11/03. doi: 10.1681/ASN.2015080922 ; PubMed Central PMCID: PMC5084889. 26961351 PMC5084889

[30] Liu X, Wu J, Zhu C, Liu J, Chen X, Zhuang T, et al. Endothelial S1pr1 regulates pressure overload-induced cardiac remodelling through AKT-eNOS pathway. J Cell Mol Med. 2019. Epub 2019/12/20. doi: 10.1111/jcmm.14900 31854513 PMC6991681

[31] Mohammed SF, Hussain S, Mirzoyev SA, Edwards WD, Maleszewski JJ, Redfield MM. Coronary microvascular rarefaction and myocardial fibrosis in heart failure with preserved ejection fraction. Circulation. 2015;131(6):550–9. Epub 2015/01/02. doi: 10.1161/CIRCULATIONAHA.114.009625 ; PubMed Central PMCID: PMC4324362. 25552356 PMC4324362

[32] Meyer M, Rambod M, LeWinter M. Pharmacological heart rate lowering in patients with a preserved ejection fraction-review of a failing concept. Heart Fail Rev. 2018;23(4):499–506. Epub 2017/11/04. doi: 10.1007/s10741-017-9660-1 ; PubMed Central PMCID: PMC5934348. 29098508 PMC5934348

[33] Ter Maaten JM, Damman K, Verhaar MC, Paulus WJ, Duncker DJ, Cheng C, et al. Connecting heart failure with preserved ejection fraction and renal dysfunction: the role of endothelial dysfunction and inflammation. Eur J Heart Fail. 2016;18(6):588–98. Epub 2016/02/11. doi: 10.1002/ejhf.497 . 26861140

[34] van Dijk CG, Oosterhuis NR, Xu YJ, Brandt M, Paulus WJ, van Heerebeek L, et al. Distinct Endothelial Cell Responses in the Heart and Kidney Microvasculature Characterize the Progression of Heart Failure With Preserved Ejection Fraction in the Obese ZSF1 Rat With Cardiorenal Metabolic Syndrome. Circ Heart Fail. 2016;9(4):e002760. Epub 2016/04/09. doi: 10.1161/CIRCHEARTFAILURE.115.002760 . 27056881

[35] Ellison DH. Treatment of Disorders of Sodium Balance in Chronic Kidney Disease. Adv Chronic Kidney Dis. 2017;24(5):332–41. Epub 2017/10/17. doi: 10.1053/j.ackd.2017.07.003 ; PubMed Central PMCID: PMC5685178. 29031361 PMC5685178

[36] Mohanan A, Gupta R, Dubey A, Jagtap V, Mandhare A, Gupta RC, et al. TRC120038, a Novel Dual AT(1)/ET(A) Receptor Blocker for Control of Hypertension, Diabetic Nephropathy, and Cardiomyopathy in ob-ZSF1 Rats. Int J Hypertens. 2011;2011:751513. Epub 2012/01/12. doi: 10.4061/2011/751513 ; PubMed Central PMCID: PMC3253485. 22235363 PMC3253485

[37] Park SH, Farooq MA, Gaertner S, Bruckert C, Qureshi AW, Lee HH, et al. Empagliflozin improved systolic blood pressure, endothelial dysfunction and heart remodeling in the metabolic syndrome ZSF1 rat. Cardiovasc Diabetol. 2020;19(1):19. Epub 2020/02/20. doi: 10.1186/s12933-020-00997-7 ; PubMed Central PMCID: PMC7026972. 32070346 PMC7026972

[38] Beldhuis IE, Streng KW, Ter Maaten JM, Voors AA, van der Meer P, Rossignol P, et al. Renin-Angiotensin System Inhibition, Worsening Renal Function, and Outcome in Heart Failure Patients With Reduced and Preserved Ejection Fraction: A Meta-Analysis of Published Study Data. Circ Heart Fail. 2017;10(2). Epub 2017/02/18. doi: 10.1161/CIRCHEARTFAILURE.116.003588 . 28209765

[39] Tofovic SP, Salah EM, Smits GJ, Whalley ET, Ticho B, Deykin A, et al. Dual A1/A2B Receptor Blockade Improves Cardiac and Renal Outcomes in a Rat Model of Heart Failure with Preserved Ejection Fraction. J Pharmacol Exp Ther. 2016;356(2):333–40. Epub 2015/11/21. doi: 10.1124/jpet.115.228841 ; PubMed Central PMCID: PMC4727158. 26585572 PMC4727158

[40] Mottram PM, Marwick TH. Assessment of diastolic function: what the general cardiologist needs to know. Heart. 2005;91(5):681–95. Epub 2005/04/16. doi: 10.1136/hrt.2003.029413 ; PubMed Central PMCID: PMC1768877. 15831663 PMC1768877

