# Supplementary figures and images for: A G-protein-biased S1P1 agonist, SAR247799, improved LVH and diastolic function in a rat model of metabolic syndrome
Source: PLoS One. 2022 Jan 14;17(1):e0257929. doi: 10.1371/journal.pone.0257929 (PMC8759645; doi:10.1371/journal.pone.0257929)

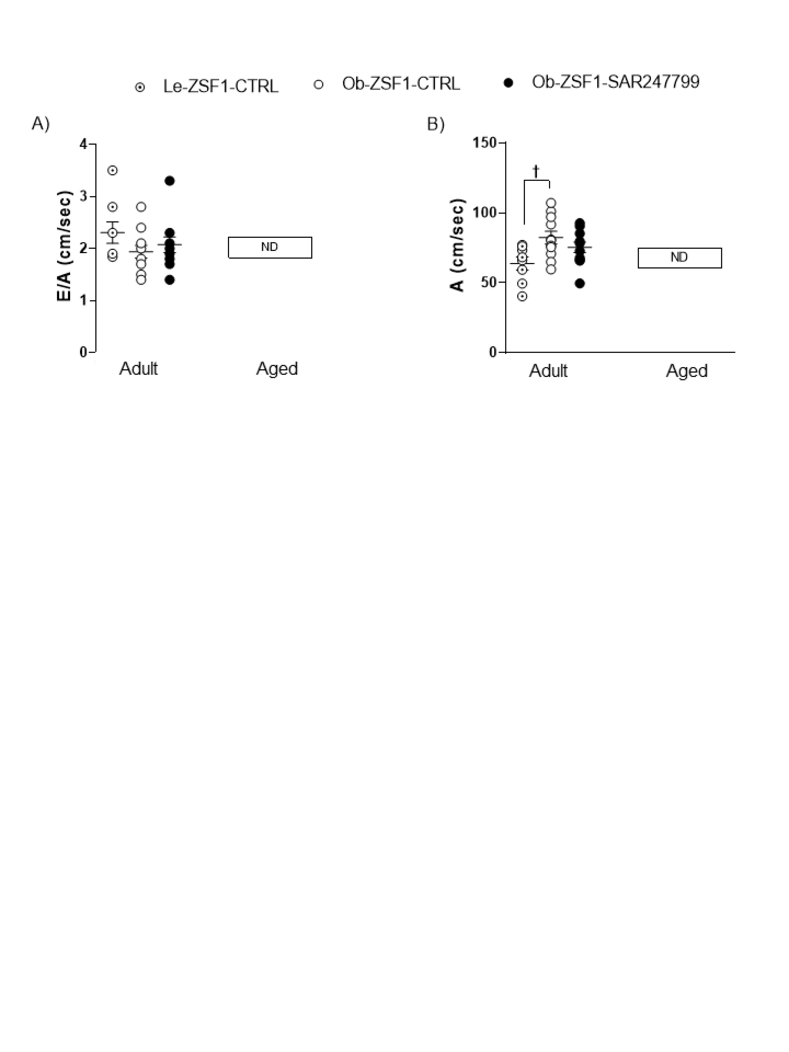

Supplement: S1 Fig — (A) E/A, (B) A wave at mitral valve. Parameters were measured in adult animals. In aged animal data were not determined (nd). Data are expressed as mean ± SEM, †p<0.05, comparison of Le-ZSF1-CTRL to Ob-ZSF1-CTRL using a Student t-test. Le-ZSF1-CTRL N = 9, Ob-ZSF1-CTRL N = 11 and Ob-ZSF1-SAR247799 N = 11. (TIF) [file pone.0257929.s001.tif]

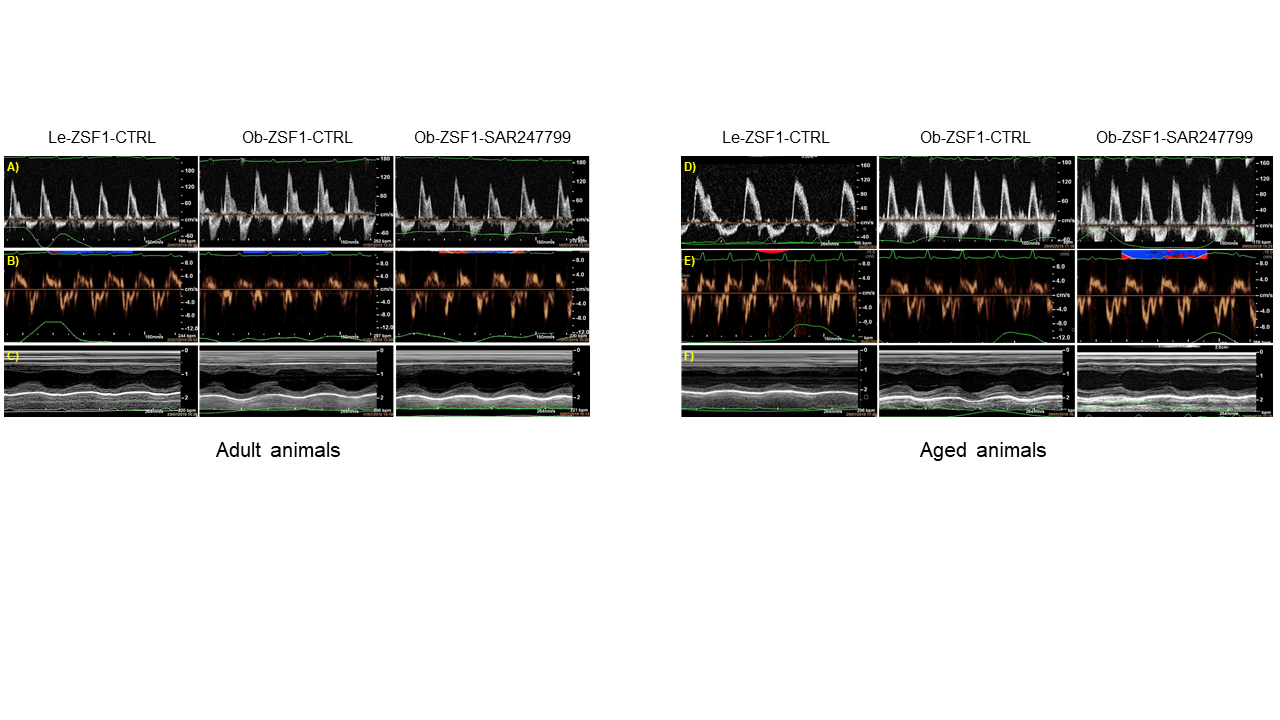

Supplement: S2 Fig — Representative echocardiographic images of E wave, e’ wave and left ventricular thickness in (A), (B) and (C) for adult animals and in (D), (E) and (F) for aged animals, respectively. (TIF) [file pone.0257929.s002.tif]
