# Supplementary material for: A G-protein-biased S1P1 agonist, SAR247799, improved LVH and diastolic function in a rat model of metabolic syndrome
Source: PLoS One. 2022 Jan 14;17(1):e0257929. doi: 10.1371/journal.pone.0257929 (PMC8759645; doi:10.1371/journal.pone.0257929)
Supplement: S1 Table — (DOCX) [file pone.0257929.s003.docx]

| **Supplementary TABLE I: Baseline cardiac parameters in adult and aged ZSF1 rats** | | | | | | | |
| --- | --- | --- | --- | --- | --- | --- | --- |
|  |  | **Adult animals** | | | **Aged animals** | | |
|  |  | **Le-ZSF1**  **(n=9)** | **Ob-ZSF1-**  ***CTRL***  **(n=11)** | **Ob-ZSF1-*SAR247799***  **(n=10-11)** | **Le-ZSF1**  **(n=9)** | **Ob-ZSF1-**  ***CTRL***  **(n=6)** | **Ob-ZSF1-*SAR247799***  **(n=6-7)** |
| **BASELINE** | IVSd  (mm) | 1.63  ± 0.125 | 1.73  ± 0.23 | 1.62  ± 0.17 | 1.73  ± 0.26 ******* | 2.24  ± 0.305 | 2.1  ± 0.41 |
|  | LVPWd  (mm) | 1.73  ± 0.17 * | 1.83  ± 0.3 | 1.82  ± 0.34 | 1.7  ± 0.33 ******* | 2.38  ± 0.427 | 2.34  ± 0.6 |
|  | Ejection fraction  % | 66.6 ± 1.50 | 68.6  ± 0.908 | 67.9  ± 1.09 | 63.1  ± 1.30 ***** | 65.8  ± 0.639 | 66.3  ± 1.20 |
|  | End-diastolic volume  (mL) | 0.618  ± 0.017 | 0.641  ± 0.018 | 0.686  ± 0.020 | 0.681  ± 0.013 ***** | 0.745  ± 0.036 | 0.757  ± 0.023 |
|  | End-systolic volume  (mL) | 0.195  ± 0.071 | 0.191  ± 0.051 | 0.232  ± 0.06 | 0.242  ± 0.043 | 0.263  ± 0.059 | 0.256  ± 0.034 |
|  | E wave  (cm/s) | 130  ± 7 | 133  ± 11 | 135  ± 18.3 | 127  ± 9 ******* | 140  ± 9.3 | 143  ± 14 |
|  | e’ wave  (cm/s) | 7.04  ± 0.5 | 6.73  ± 0.46 | 6.72  ± 0.32 | 7.47  ± 0.67 | 6.84  ± 0.995 | 6.99  ± 1.41 |
|  | E/e’ | 18.8  ± 1.2 ***** | 20.2  ± 1.2 | 20.5  ± 4.4 | 16.9  ± 1.2 ******* | 20.3  ± 2.77 | 20.5  ± 3.3 |
|  | Stroke volume  (mL) | 0.395  ± 0.082 ****** | 0.506  ± 0.121 | 0.552  ± 0.049 | 0.358  ± 0.11 ******* | 0.564  ± 0.058 | 0.506  ± 0.08 |
|  | Cardiac index  (mL/min/cm^2^) | 0.202  ± 0.032 | 0.206  ± 0.05 | 0.222  ± 0.025 | 0.169  ± 0.032 | 0.191  ± 0.02 | 0.172  ± 0.069 |
| Interventricular septum at end-diastole (IVSd), left ventricular posterior wall at end-diastole (LVPWd), ejection fraction, end-diastolic volume, end-systolic volume, stroke volume, cardiac index.  Data are expressed as mean ± SEM, except IVSd, LVPWd, End-diastolic volume, E wave, e’ wave, E/e’, stroke volume and cardiac index who are expressed as median ± IQR.  *****p<0.05, ******p<0.01, *******p<0.001: p-values obtained to compare Le-ZSF1 to Ob-ZSF1 (Ob-ZSF1-CTRL and Ob-ZSF1-SAR247799 pooled) using a Student t-test except for IVSd, stroke volume and cardiac index parameters for adult animals and for end-systolic volume for aged animals for which a Wilcoxon test was performed. A Student t-test or Wilcoxon test, when appropriate, was also performed to compare Ob-ZSF1-CTRL to Ob-ZSF1-SAR247799 at baseline. No significant difference was observed between the two Ob-ZSF1 groups at baseline for any of the parameters. A lower number of n values is due to technical issue in echocardiography data collection. | | | | | | | |
