# Supplementary material for: A G-protein-biased S1P1 agonist, SAR247799, improved LVH and diastolic function in a rat model of metabolic syndrome
Source: PLoS One. 2022 Jan 14;17(1):e0257929. doi: 10.1371/journal.pone.0257929 (PMC8759645; doi:10.1371/journal.pone.0257929)
Supplement: S2 Table — A) Daily food intake at two weeks after treatment in adult and aged rats; B) Individual value of daily food intake and SAR247799 plasma concentration. (DOCX) [file pone.0257929.s004.docx]

**Supplementary TABLE II: Daily food intake and SAR247799 plasma concentration**

**A) Daily food intake at two weeks after treatment in adult and aged rats**

|  | **Adult animals** | | | **Aged animals** | | |
| --- | --- | --- | --- | --- | --- | --- |
|  | **Le-ZSF1**  **CTRL**  **(n=9)** | **Ob-ZSF1-**  ***CTRL***  **(n=11)** | **Ob-ZSF1-*SAR247799***  **(n=10-11)** | **Le-ZSF1**  **CTRL**  **(n=9)** | **Ob-ZSF1-**  ***CTRL***  **(n=6)** | **Ob-ZSF1-*SAR247799***  **(n=6-7)** |
| Daily food intake (g) | 29.1 ± 1.13 | 48.6 ± 1.12 **^†††^** | 47.9 ± 1.67 | 26.5 ± 2.35 | 26.8 ± 2.52 | 27.2 ± 1.14 |
| Daily food intake measured 2 weeks after the beginning of treatment with CTRL or SAR247799.  Data are expressed as mean ± SEM  **†††** p<0.001 from the comparison between Le-ZSF1-CTRL and Ob-ZSF1-CTRL using a Student t-test. | | | | | | |

**B) Individual value of daily food intake and SAR247799 plasma concentration**

| **Adult animals with LVH and diastolic dysfunction** | | |
| --- | --- | --- |
| **Animal** | **Daily food intake (g)** | **SAR247799**  **Plasma concentration (ng/mL)** |
| 3711 | nd | 2360 |
| 3717 | 47 | 4020 |
| 3810 | 52 | 2730 |
| 3830 | 52 | 3060 |
| 5063 | 52 | 2280 |
| 5068 | 52 | 4810 |
| 5095 | 44 | 4090 |
| 5108 | 46 | 2680 |
| 5219 | 47 | 2820 |
| 6034 | 44 | 6530 |
| 6042 | 45 | 4520 |
| **Aged animals with LVH and diastolic dysfunction** | | |
| **Animal** | **Daily food intake (g)** | **SAR247799**  **Plasma concentration (ng/mL)** |
| 8876 | 31 | 567 |
| 8913 | 26 | 667 |
| 8989 | 29 | 448 |
| 8990 | 31 | 1110 |
| 8992 | 29 | 431 |
| 9002 | 23 | 110 |
| 9025 | 23 | 198 |
| Individual values of daily food intake of Ob-ZSF1-SAR247799 rats and corresponding SAR247799 plasma exposure. Daily food intake data here shown was recorded one week before blood collection for SAR247799 plasma evaluation. | | |
